# Distributed Mode-Dependent Event-Triggered Passive Filtering for Flexible Manipulator with Semi-Markov Parameters

**DOI:** 10.3390/s21062058

**Published:** 2021-03-15

**Authors:** Yidao Ji, Suiwu Zheng

**Affiliations:** 1 School of Automation and Electrical Engineering, University of Science and Technology Beijing, Beijing 100083, China; yidao.ji@xs.ustb.edu.cn; 2 Institute of Automation, Chinese Academy of Sciences, Beijing 100190, China; 3 Huizhou Zhongke Advanced Manufacturing Research Center Co., Ltd., Huizhou 516000, China

**Keywords:** distributed filtering, flexible manipulator, event-triggered filtering, semi-Markov parameters

## Abstract

In this paper, the passive filtering problem of flexible robotic manipulator is investigated over sensor networks in a distributed manner from the control system perspective. The sensor networks are adopted to estimate true states of flexible robotic manipulator. In particular, the semi-Markov model is utilized for flexible manipulators with varying loads in unstructured environment, which is more flexible for practical implementations. Moreover, the new mode-dependent event-triggering mechanism is developed for distributed filter communications. Based on model transformation, sufficient conditions are first established to guarantee prescribed passive performance under disturbances. Then, desired mode-dependent filters are developed with the aid of convex optimization. In the end, several simulations results of a single-link flexible robotic manipulator are provided to verify the usefulness of the developed filtering algorithm.

## 1. Introduction

In the recent years, robotic manipulators have been more and more widely applied in various fields, such as industrial manufacturing [1], agricultural production [2,3], space exploration [4], etc. Especially, flexible robotic manipulators have attracted a huge attention by scientists and engineers since their distinguishing advantages, which include multi-degree of freedom, changeable structures, higher efficient payload and lower energy consumption [5,6,7]. Meanwhile, the manipulation tasks of robotic manipulators are becoming more and more complex due to practical demands. For instance, one interesting problem is how to achieve stable control of robotic manipulators with payloads of different weights in unstructured working environment. Furthermore, it should be pointed out that in certain tasks the changing of payloads could exhibit random features. Thus, some efforts have been made by applying the hybrid system models. To name a few, in [8], the trajectory control issue of robotic manipulators based on Radial Basis Function (RBF) neural networks is studied by the formulated hybrid switched system model. Furthermore, in [9], multiple models for robotic manipulator are discussed for the adaptive control problem. Besides, the load shifting robot manipulator model is investigated in [10]. It is worth mentioning that Markov jump systems are always used to model complex systems with parameter jumping or different modes, while the jumping modes and the transition probabilities between systems’ modes could be depicted accordingly [11,12,13]. As a result, it is reasonable to describe the flexible robotic manipulators with varying loads by Markov jump models. In addition, it is worth mentioning that the transition probabilities of Markov jump systems are based on fixed exponential distribution, which has ceratin restrictions in practical applications. In fact, the transition probabilities could be time-varying, which gives rises to the researches on so-called semi-Markov jump systems [14,15,16]. As such, semi-Markov jump systems have been intensively studied recently by researchers and various remarkable results on analysis and synthesis problems of semi-Markov jump systems have been reported in the literature. Therefore, it is important to study the flexible robotic manipulators with semi-Markov parameters.

On another active research area, with the substantial development of sensor and network technologies, sensor networks have been extensively studied in recent years, which means that a group of sensors are working collaboratively and changing information with each other. Compared with traditional single sensor, sensor networks can have many working merits, such as better robustness, higher efficiency, lower cost and so on. As one of the fundamental yet significant problems of sensor networks, the state estimation and filtering issues based on sensor networks have been widely studied to estimate the true values of certain states in target systems. In particular, various distributed filtering algorithms over sensor networks have been developed in recent years. Well known examples can be found by distributed $H_{\infty }$ filtering, passivity filtering, dissipative filtering approaches and so on [17,18,19]. For instance, in [20], distributed $H_{\infty }$ filtering is carried out over lossy sensor networks and dissipative filtering with missing measurements is investigated in [21]. Especially, the passivity performance has been proven to be effective for filtering problem from energy input-output perspective [22]. Very recently, by noticing that the common time-triggered strategies of distributed filtering need considerable communication consumption, the novel event-triggered strategies have been proposed to cope with the information exchanges among the sensors [23,24,25]. More precisely, the event-triggered strategies accomplish the signal transmission by certain prescribed event conditions instead of data transmission according to a period of time while a satisfactory task performance can still be ensured. For the distributed filtering of semi-Markov flexible robotic manipulators with diverse modes, the jumping mode information should be used to further reduce the conservatism. However, to the authors’ best knowledge, up to now, there is still little concern on the distributed filtering issues of semi-Markov jump systems within mode-dependent event-triggered framework, which remains an open and challenging problem in this research field.

Motivated by the aforementioned discussions, in this paper, the distributed mode-dependent event-triggered passive filtering algorithm is addressed for semi-Markov flexible manipulators. In comparison of most reported literature, our contributions are made towards the general filtering problem of semi-Markov jump systems with potential applications to flexible robotic manipulators, which can be concluded as follows:

(1) A new distributed mode-dependent event-triggering filtering algorithm is developed over sensor networks, which is based on the formulated semi-Markovian flexible manipulator, which can further extend the event-triggered strategy and can well utilize system mode information. The developed filtering algorithm is with asynchronous sampled-data and is thus more general and applicable in the practical applications.

(2) As an alternative to common $H_{\infty }$ filtering method, the concept of passivity performance is adopted from an energy input-output perspective. By designing the distributed passive filter, the desired passivity performance can be achieved with external disturbance attenuation.

(3) On the basis of convex optimization and Lyapunov–Krasovskii methods, the mode-dependent filter gains and event-triggering parameters are designed simultaneously. At last, the effectiveness of our proposed filtering algorithm is demonstrated via a numerical example.

The remainder structure of our paper would be organized by the following parts. Section 2 formulates the distributed filtering problem of semi-Markovian flexible manipulator while mode-dependent event-triggered filter design is introduced. Section 3 presents the primary design method by proven details. Section 4 demonstrates our proposed algorithm by numerical simulations. In the final section, the paper is concluded and future research prospect is given.

The following notations are standardly used through the paper. $R^{n}$ denotes the *n* dimensional Euclidean space. Matrix P>0 implies that *P* is positive definite. diag{⋯} denotes the block-diagonal matrix. E() represents the expectation operator. $L_{2}\left[0,\infty \right)$ represents space of square-integrable vector functions over [0,∞).

## 2. Preliminaries and Problem Formulation

### 2.1. Flexible Manipulator with Semi-Markov Parameters

Since flexible robotic manipulators always manipulate varying loads in unstructured environment, it is flexible and reasonable to model the parameter changing features caused by varying loads for practical implementations. For some applications, the load shifting can be modeled by semi-Markov chains, such that the parameter changing of flexible robotic manipulators can be conducted by semi-Markov chains accordingly.

In order to describe the semi-Markov chain, give a probability space (O,F,P), where O denotes sample space, F represents δ-algebra of subsets of O and P stands for probability measure on F. Let {σ(t),t≥0} denote a continuous-time discrete-state semi-Markov process on taking values in a finite set S={1,…,N}. Furthermore, the transition probability matrix $\Pi :=(\pi_{ij}\left(h\right))$, h>0, ∀i,j∈I is defined in Equation (1)

(1) $$\mathrm{Pr}\left(\sigma \left(t+h\right)=j|\sigma \left(t\right)=i\right)=\left\{\begin{array}{c} \pi_{ij}\left(h\right)h+o\left(h\right),\;\;i\neq j,\\ 1+\pi_{ii}\left(h\right)h+o\left(h\right),\;\;i=j, \end{array}\right.$$

where lim(o(h)/h)=0 with o(h) denoting weak infinitesimal generator, $\pi_{ij}\left(h\right)\geq 0$, i≠j, is the transition rate from mode *i* at time *t* to mode *j* at time t+h, satisfying $\pi_{ii}\left(h\right)=-$$\sum_{j=1,\;j\neq i}^{N}\pi_{ij}\left(h\right)$, ∀i∈S.

Consider the following flexible-link manipulator with the semi-Markov jumping dynamics and σ(t) denotes the semi-Markov chain in (O,F,P), which is defined in Equation (2):

(2) $$\left\{\begin{array}{c} {\dot{\theta }}_{m}=\omega_{m},\\ {\dot{\omega }}_{m}=\frac{k}{J_{m}\left(\sigma \left(t\right)\right)}\left(\theta_{l}-\theta_{m}\right)-\frac{B}{J_{m}\left(\sigma \left(t\right)\right)}\omega_{m}+\frac{1}{J_{m}\left(\sigma \left(t\right)\right)}u,\\ {\dot{\theta }}_{l}=\omega_{l},\\ {\dot{\omega }}_{l}=-\frac{k}{J_{l}\left(\sigma \left(t\right)\right)}\left(\theta_{l}-\theta_{m}\right)-\frac{m(\sigma (t))gh}{J_{l}\left(\sigma \left(t\right)\right)}\sin\left(\theta_{l}\right), \end{array}\right.$$

where $J_{m}\left(\sigma \left(t\right)\right)$ denotes the inertia of motor, $J_{l}\left(\sigma \left(t\right)\right)$ denotes the inertia of link, $\theta_{m}$ denotes the angular rotation of motor, $\theta_{l}$ denotes the angular position of link, $\omega_{m}$ denotes the angular velocity of motor, *k* denotes the joint elastic constant, m(σ(t)) denotes the link mass, *l* denotes the link length, *g* is the gravity constant, *B* is the viscosity, $\omega_{l}$ denotes the angular velocity of link. More details of the parameters can be found in the literature [26].

Furthermore, by assuming certain *u* that can stabilize the manipulator and taking into account the external disturbance $w_{1}\left(t\right)\in L_{2}\left[0,\infty \right)$, the following state-space nonlinear model can be obtained in Equation (3):

(3) $$\left\{\begin{array}{c} \dot{x}\left(t\right)=A\left(\sigma \left(t\right)\right)x\left(t\right)+g\left(\sigma \left(t\right),x\left(t\right)\right)+B\left(\sigma \left(t\right)\right)w_{1}\left(t\right)\\ z(t)=E(\sigma (t))x(t) \end{array}\right.$$

where $x\left(t\right)={\left[\theta_{m},\omega_{m},\theta_{1},\omega_{1}\right]}^{T}$ represents formulated manipulator system state, z(t) represents the output to be estimated over sensor network, w(t) denotes the disturbance and A(σ(t)), B(σ(t)), E(σ(t)) are defined in Equations (4)–(7)

(4) $$\begin{array}{cc} A(\sigma (t))= & \left[\begin{array}{cccc} 0 & 1 & 0 & 0\\ -\frac{k}{J_{m}\left(\sigma \left(t\right)\right)} & -\frac{B}{J_{m}\left(\sigma \left(t\right)\right)} & \frac{k}{J_{m}\left(\sigma \left(t\right)\right)} & 0\\ 0 & 0 & 0 & 1\\ \frac{k}{J_{l}\left(\sigma \left(t\right)\right)} & 0 & -\frac{k}{J_{l}\left(\sigma \left(t\right)\right)} & 0 \end{array}\right], \end{array}$$

(5) $$\begin{array}{cc} g(\sigma (t),x(t)) & =\left[\begin{array}{c} 0\\ 0\\ 0\\ -\frac{m(\sigma (t))gh}{J_{1}\left(\sigma \left(t\right)\right)}\sin\left(\theta_{1}\right) \end{array}\right], \end{array}$$

(6) $$\begin{array}{cc} B(\sigma (t)) & =\left[\begin{array}{c} 0\\ \frac{1}{J_{m}\left(\sigma \left(t\right)\right)}\\ 0\\ 0 \end{array}\right], \end{array}$$

(7) $$\begin{array}{cc} E(\sigma (t)) & =\left[\begin{array}{cccc} 1 & 0 & 0 & 0\\ 0 & 1 & 0 & 0 \end{array}\right]. \end{array}$$

Moreover, it follows that g(σ(t),x(t)) is a nonlinear function and can satisfy the Lipschitz nonlinear conditions with $\iota \left(\sigma \left(t\right)\right)=-\frac{m(\sigma (t))gh}{J_{1}}$.

**Remark** **1.** *The formulated state-space model can be generally utilized for nonlinear semi-Markov jump systems with Lipschitz conditions. Under this context, the distributed filtering problem can be further solved by convex optimization design.*

### 2.2. Distributed Filter Design over Sensor Networks

For the sensor network, a direct graph $G=\left\{V,E,A\right\}$ is presented to describe the communication topology of *N* sensor nodes, where $V=\left\{v_{1},v_{2},\cdots ,v_{N}\right\}$ and E⊆V×V stand for the set of nodes and edges, respectively, $A=\left[a_{ij}\right]\in R^{N\times N}$ represent the adjacency matrix with $a_{ii}=0$ for any *i*. A is associated with the edges of G are positive, i.e., $a_{ij}>0⟺\varepsilon_{ij}\in E$. In addition, the corresponding Laplacian matrix of G is defined as L.

**Figure 1:**
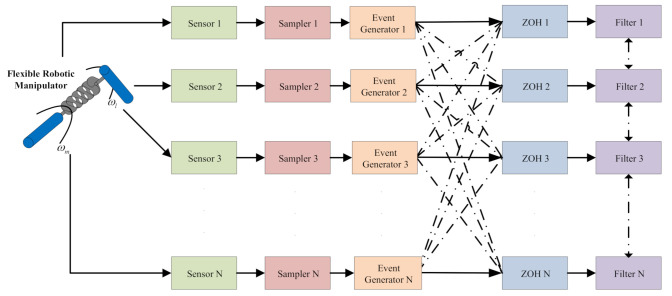
Illustration of distributed filtering on sensor network.

As depicted in Figure 1, it is supposed that all the sampler of sensors are time-driven with sampling instants $t_{k}$ and zero-order-holders are event-driven. The sampling period is set by $h_{k}=t_{k+1}-t_{k}\leq \bar{d}$. The measured output of sensor *m* can be obtained in Equation (8):

(8) $$y_{m}\left(t\right)=C_{m}\left(\sigma \left(t\right)\right)x\left(t\right)+D_{m}\left(\sigma \left(t\right)\right)w_{2}\left(t\right),m=1,2,\ldots ,N,$$

where $y_{m}\left(t\right)$ represents the measured output of sensor *m*, $C_{m}\left(\sigma \left(t\right)\right)\in R^{q}$ and $D_{m}\left(\sigma \left(t\right)\right)\in R^{m}$ are known constant matrices, $w_{2}\left(t\right)$ stands for the disturbance over sensors belonging to $L_{2}\left[0,\infty \right)$. Moreover, the event-triggered generator with event condition is deployed to transmit the latest data.

Consequently, denote ${\hat{x}}_{m}\left(t\right)$ as the estimated value of x(t) by sensor *m* and the sensor estimation error ${\tilde{y}}_{m}\left(t\right)$ can be obtained in Equation (9):

(9) $${\tilde{y}}_{m}\left(t\right)=y_{m}\left(t\right)-C_{m}\left(\sigma \left(t\right)\right){\hat{x}}_{m}\left(t\right).\;$$

Then, ${\tilde{y}}_{m}\left(t_{k}\right)$ is sampled at each $t_{k}$ and ${\tilde{y}}_{m}\left(t_{\delta }^{m}\right)$ is transmitted according to the network topology when the event-triggering condition is satisfied. Besides, the broadcasting instant $t_{\delta +1}^{m}$ of sensor *m* satisfies the following event condition in Equation (10):

(10) $$t_{\delta +1}^{i}=\min_{t_{k}>t_{\delta }^{m}}\left\{t_{k}∥{\tilde{y}}_{m}\left(t_{k}\right)-{\tilde{y}}_{m}\left(t_{\delta }^{m}\right)∥\geq \kappa_{m}\left(\sigma \left(t\right)\right)∥{\tilde{y}}_{m}\left(t_{\delta }^{m}\right)∥\right\},$$

where $0<\kappa_{m}\left(\sigma \left(t\right)\right)<1$ denotes the threshold parameter.

**Remark** **2.** *It can be found that our proposed event-triggered strategy can lead to asynchronous local information exchanges between the sensors, which is more practical and applicable for distributed sensor networks.*

As such, the distributed mode-dependent filter can be designed in Equation (11):

(11) $$\begin{array}{c} \dot{{\hat{x}}_{m}}\left(t\right)=A\left(\sigma \left(t\right)\right){\hat{x}}_{m}\left(t\right)+g\left(\sigma \left(t\right),{\hat{x}}_{m}\left(t\right)\right)+F_{m}\left(\sigma \left(t\right)\right){\tilde{y}}_{m}\left(t_{\delta }^{m}\right)+K_{m}\left(\sigma \left(t\right)\right)\sum_{m=1}^{N}a_{mn}\left[{\tilde{y}}_{n}\left(t_{\delta_{m}^{{}^{\prime }}\left(t\right)}^{j}\right)-{\tilde{y}}_{m}\left(t_{\delta }^{m}\right)\right],\\ {\hat{z}}_{m}\left(t\right)=E\left(\sigma \left(t\right)\right){\hat{x}}_{m}\left(t\right),\;\;\;t\in \left[t_{\delta }^{m},t_{\delta +1}^{m}\right),m_{j}^{{}^{\prime }}\left(t\right)=\arg\min_{p}\left\{t-t_{p}^{m}|t\geq t_{p}^{m}\right\}. \end{array}$$

where ${\hat{z}}_{m}\left(t\right)\;$ denotes the estimate of z(t), $F_{m}\left(\sigma \left(t\right)\right)$ and $K_{m}\left(\sigma \left(t\right)\right)$ denotes mode-dependent filter gains to be designed.

By defining the sampled-data error in Equation (12):

(12) $$\epsilon_{m}\left(s_{k}\right)={\tilde{y}}_{m}\left(t_{k}\right)-{\tilde{y}}_{m}\left(t_{\delta }^{m}\right),\;\;t_{\delta }^{m}\leq t_{k}<t_{\delta +1}^{m},$$

and dividing the interval $\left[t_{\delta }^{m},t_{\delta +1}^{m}\right)$ with $\cup_{t_{k}=t_{\delta }^{m}}^{t_{\delta +1}^{m}-h_{k+1}}\left[t_{k},t_{k+1}\right)$, it can be derived in Equation (13) that

(13) $$\left\{\begin{array}{c} \dot{{\hat{x}}_{m}}\left(t\right)=A\left(\sigma \left(t\right)\right){\hat{x}}_{m}\left(t\right)+g\left(\sigma \left(t\right),{\hat{x}}_{m}\left(t\right)\right)+F_{m}\left(\sigma \left(t\right)\right){\tilde{y}}_{m}\left(s_{k}\right)+K_{m}\left(\sigma \left(t\right)\right)\sum_{n=1}^{N}a_{mn}\left[{\tilde{y}}_{n}\left(t_{k}\right)-{\tilde{y}}_{m}\left(t_{k}\right)\right]\\ \left.\right.\left.\right.\left.\right.\left.\right.\left.\right.\left.\right.\left.\right.\left.\right.\left.\right.\left.\right.-F_{m}\left(\sigma \left(t\right)\right)\epsilon_{m}\left(t_{k}\right)-K_{m}\left(\sigma \left(t\right)\right)\sum_{j=1}^{N}a_{mn}\left[\epsilon_{n}\left(t_{k}\right)-\epsilon_{m}\left(t_{k}\right)\right],\\ {\hat{z}}_{m}\left(t\right)=E\left(\sigma \left(t\right)\right){\hat{x}}_{m}\left(t\right),\;\;\;t\in \left[t_{k},t_{k+1}\right). \end{array}\right.$$

As a result, by letting $e_{m}\left(t\right)=x\left(t\right)-{\hat{x}}_{m}\left(t\right)$ and ${\tilde{z}}_{m}\left(t\right)=z\left(t\right)-{\hat{z}}_{m}\left(t\right)$, the filtering error dynamics can be obtained in Equation (14):

(14) $$\left\{\begin{array}{cc} {\dot{e}}_{m}\left(t\right) & =A\left(\sigma \left(t\right)\right)e_{m}\left(t\right)+g\left(\sigma \left(t\right),x\left(t\right)\right)-g\left(\sigma \left(t\right),{\hat{x}}_{m}\left(t\right)\right)-F_{m}\left(\sigma \left(t\right)\right)C_{m}\left(\sigma \left(t\right)\right)e_{m}\left(t_{k}\right)\\ & -K_{m}\left(\sigma \left(t\right)\right)\sum_{n=1}^{N}a_{mn}\left[C_{n}\left(\sigma \left(t\right)\right)e_{n}\left(t_{k}\right)-C_{m}\left(\sigma \left(t\right)\right)e_{m}\left(t_{k}\right)\right]\\ & +F_{m}\left(\sigma \left(t\right)\right)\epsilon_{m}\left(t_{k}\right)+K_{m}\left(\sigma \left(t\right)\right)\sum_{m=1}^{N}a_{mn}\left[\epsilon_{j}\left(t_{k}\right)-\epsilon_{i}\left(t_{k}\right)\right]+B\left(\sigma \left(t\right)\right)w_{1}\left(t\right)-F_{m}\left(\sigma \left(t\right)\right)D_{m}\left(\sigma \left(t\right)\right)w_{2}\left(t_{k}\right)\\ & -K_{m}\left(\sigma \left(t\right)\right)\sum_{n=1}^{N}a_{mn}\left[D_{n}\left(\sigma \left(t\right)\right)v_{2}\left(t_{k}\right)-D_{m}\left(\sigma \left(t\right)\right)v_{2}\left(t_{k}\right)\right],\\ {\tilde{z}}_{m}\left(t\right) & =E\left(\sigma \left(t\right)\right)e_{m}\left(t\right),\;t\in \left[t_{k},t_{k+1}\right), \end{array}\right.$$

which can be further rewritten in Equation (15):

(15) $$\left\{\begin{array}{cc} \dot{e}\left(t\right) & =\bar{A}\left(\sigma \left(t\right)\right)e\left(t\right)+G\left(\sigma \left(t\right),e\left(t\right)\right)-\left(F\left(\sigma \left(t\right)\right)-K\left(\sigma \left(t\right)\right)L\left(\sigma \left(t\right)\right)\right)C\left(\sigma \left(t\right)\right)e\left(t_{k}\right)\\ & +\left(F\left(\sigma \left(t\right)\right)-K\left(\sigma \left(t\right)\right)L\left(\sigma \left(t\right)\right)\right)\epsilon \left(t_{k}\right)+\bar{B}\left(\sigma \left(t\right)\right)w_{1}\left(t\right)\\ & -\left(F\left(\sigma \left(t\right)\right)-K\left(\sigma \left(t\right)\right)L\left(\sigma \left(t\right)\right)\right)\bar{D}\left(\sigma \left(t\right)\right)w_{2}\left(t_{k}\right),\\ \tilde{z}\left(t\right) & =\bar{E}\left(\sigma \left(t\right)\right)e\left(t\right),\;\;\;t\in \left[t_{k},t_{k+1}\right) \end{array}\right.$$

where

$$\begin{array}{cc} e(t)= & {\left[e_{1}^{T}\left(t\right),e_{2}^{T}\left(t\right),\ldots ,e_{N}^{T}\left(t\right)\right]}^{T},\\ \tilde{z}\left(t\right)= & {\left[{\tilde{z}}_{1}^{T}\left(t\right),{\tilde{z}}_{2}^{T}\left(t\right),\ldots ,{\tilde{z}}_{N}^{T}\left(t\right)\right]}^{T},\\ \epsilon (t)= & {\left[\epsilon_{1}^{T}\left(t\right),\epsilon_{2}^{T}\left(t\right),\ldots ,\epsilon_{N}^{T}\left(t\right)\right]}^{T},\\ \bar{A}\left(\sigma \left(t\right)\right)= & I_{N}⊗A\left(\sigma \left(t\right)\right),\\ G(\sigma (t),e(t))= & [{\left(g\left(\sigma \left(t\right),x\left(t\right)\right)-g\left(\sigma \left(t\right),{\hat{x}}_{1}\left(t\right)\right)\right)}^{T},{\left(g\left(\sigma \left(t\right),x\left(t\right)\right)-g\left(\sigma \left(t\right),{\hat{x}}_{2}\left(t\right)\right)\right)}^{T},\\ \; & \ldots {\left(g\left(\sigma \left(t\right),x\left(t\right)\right)-g\left(\sigma \left(t\right),{\hat{x}}_{N}\left(t\right)\right)\right)}^{T}{]}^{T},\\ C(\sigma (t))= & diag\left\{C_{i}\left(\sigma \left(t\right)\right)\right\},\\ F(\sigma (t))= & diag\left\{F_{i}\left(\sigma \left(t\right)\right)\right\},\\ K(\sigma (t))= & diag\left\{K_{i}\left(\sigma \left(t\right)\right)\right\},\\ L(\sigma (t))= & L(\sigma (t))⊗I,\\ \bar{B}\left(\sigma \left(t\right)\right)= & 1⊗B(\sigma (t)),\\ \bar{D}\left(\sigma \left(t\right)\right)= & {\left[D_{1}^{T}\left(\sigma \left(t\right)\right),D_{2}^{T}\left(\sigma \left(t\right)\right),\ldots ,D_{N}^{T}\left(\sigma \left(t\right)\right)\right]}^{T},\\ \bar{E}\left(\sigma \left(t\right)\right)= & I⊗E(\sigma (t)). \end{array}$$

Moreover, it can be verified that $G{\left(\sigma \left(t\right)\right)}^{T}\left(e\left(t\right)\right)G\left(\sigma \left(t\right)\right)\left(e\left(t\right)\right)\leq \Gamma \left(\sigma \left(t\right)\right)e^{T}\left(t\right)e\left(t\right)$, $\Gamma \left(\sigma \left(t\right)\right)=diag\left\{\iota (\sigma (t)),\iota (\sigma (t)),\ldots ,\iota (\sigma (t))\right\}.$

### 2.3. Filtering Objective

For denoting simplicity, denote mode σ(t) as *i* index and employ the novel input-delay method. Then, the overall filtering error dynamics can be deduced in Equation (16):

(16) $$\left\{\begin{array}{cc} \dot{e}\left(t\right) & ={\bar{A}}_{i}e\left(t\right)+G_{i}\left(e\left(t\right)\right)-\left(F_{i}-K_{i}L_{i}\right)C_{i}e\left(t-d\left(t\right)\right)\\ & +\left(F_{i}-K_{i}L_{i}\right)\epsilon \left(t-d\left(t\right)\right)+{\bar{B}}_{i}w_{1}\left(t\right)-\left(F_{i}-K_{i}L_{i}\right){\bar{D}}_{i}w_{2}\left(t-d(t)\right),\\ \tilde{z}\left(t\right) & ={\bar{E}}_{i}e\left(t\right),t\in \left[t_{k},t_{k+1}\right) \end{array}\right.$$

where $d\left(t\right)=t-t_{k}$, $t\in \left[t_{k},t_{k+1}\right)$ with $0\leq d\left(t\right)<\bar{d}$.

Before proceeding further, the following passivity performance index in Equation (17) is introduced for the distributed filters.

**Definition** **1.** *Under the zero-initial condition, the modified passivity performance γ is said to be achieved in the mean-square sense, if it holds that*

(17) $$2E\left\{\int_{0}^{T}{\tilde{z}}^{T}\left(t\right)S\left(w_{1}\left(t\right)+w_{2}\left(t-d(t)\right)\right)dt\right\}\geq -\gamma \int_{0}^{T}w_{1}^{T}\left(t\right)w_{1}\left(t\right)+w_{2}^{T}\left(t-d(t)\right)w_{2}\left(t-d(t)\right)dt,$$

*where matrix S is with appropriate dimension.*

The control objective is to design the desired filter gains $K_{i}$ and $F_{i}$ for all filters, such that the passivity performance can be achieved according to Definition 1.

The following useful lemma is given for later derivations.

**Lemma** **1.** *Ref. [27] For any matrix M>0, scalars τ>0, τ(t) satisfying 0≤τ(t)≤τ, vector function $\dot{x}\left(t\right):\left[-\tau ,0\right]\to R^{n}$ such that the concerned integrations are well defined in Equation (18), then*

(18) $$-\tau \int_{t-\tau }^{t}{\dot{x}}^{T}\left(s\right)M\dot{x}\left(s\right)ds\leq \zeta^{T}\left(t\right)U\zeta \left(t\right),$$

*where*

$$\begin{array}{cc} \zeta (t)= & {\left[x^{T}\left(t\right),x^{T}\left(t-\tau \left(t\right)\right),x^{T}\left(t-\tau \right)\right]}^{T},\\ U= & \left[\begin{array}{ccc} -M & M & 0\\ {}* & -2M & M\\ {}* & * & -M \end{array}\right]. \end{array}$$

## 3. Distributed Filtering Analysis and Filter Gain Design

In this section, the mode-dependent distributed filters are designed with proven details.

**Theorem** **1.** *For given $h_{k}$ and mode-dependent filter gains $K_{i}$ and $F_{i}$, the passivity filtering can be achieved according to Definition 1, if there exist mode-dependent matrices $P_{i}>0$, mode-dependent parameters $\kappa_{i}$, and matrices Q>0, R>0, such that $\Xi_{i,k}<0$, for all i∈N, k=1,2,…,K, where*

$$\begin{array}{cc} \Xi_{i,k}: & =\left[\begin{array}{cc} \Xi_{1i,k} & \Xi_{2i,k}\\ {}* & \Xi_{3i,k} \end{array}\right],\\ \Xi_{1i,k}: & =\left[\begin{array}{cccc} 2P_{i}{\bar{A}}_{i}+Q-R+\Gamma_{i}+\sum_{j=1}^{N}\pi_{ij,k}P_{j} & -P_{i}F_{i}C_{i}+P_{i}K_{i}L_{i}C_{i}+R & 0 & P_{i}F_{i}-P_{i}K_{i}L_{i}\\ {}* & -2R+C^{T}\left(\Lambda ⊗I_{q}\right)C & R & -C^{T}\left(\Lambda ⊗I_{q}\right)\\ {}* & * & -Q-R & 0\\ {}* & * & * & \left(\Lambda -I_{N}\right)⊗I_{q} \end{array}\right],\\ \Xi_{2i,k}: & =\left[\begin{array}{cccc} P_{i} & P_{i}{\bar{B}}_{i}-{\bar{E}}_{i}^{T} & -P_{i}F_{i}{\bar{D}}_{i}+P_{i}K_{i}L_{i}{\bar{D}}_{i}-{\bar{E}}_{i}^{T}S & \bar{d}{\bar{A}}_{i}^{T}R\\ 0 & 0 & 0 & -\bar{d}C_{i}^{T}F_{i}^{T}R+\bar{d}C_{i}^{T}L_{i}^{T}K_{i}^{T}R\\ 0 & 0 & 0 & 0\\ 0 & 0 & 0 & \bar{d}F_{i}^{T}R-\bar{d}L_{i}^{T}K_{i}^{T}R \end{array}\right],\\ \Xi_{3i,k}: & =\left[\begin{array}{cccc} -I & 0 & 0 & \bar{d}R\\ {}* & -\gamma I & 0 & \bar{d}{\bar{B}}_{i}^{T}R\\ {}* & * & -\gamma I & -\bar{d}{\bar{D}}_{i}^{T}F_{i}^{T}R+\bar{d}{\bar{D}}_{i}^{T}L_{i}^{T}K_{i}^{T}R\\ {}* & * & * & -R \end{array}\right]. \end{array}$$

**Proof.**  For each mode *i*, the mode-dependent Lyapunov-Krasovskii functionals are constructed in Equation (19):

(19) $$V\left(i,t\right)=V_{1}\left(i,t\right)+V_{2}\left(i,t\right)+V_{3}\left(i,t\right),$$

where

$$\begin{array}{cc} V_{1}\left(i,t\right)= & e^{T}\left(t\right)P_{i}e\left(t\right),\\ V_{2}\left(i,t\right)= & \int_{t-\bar{d}}^{t}e^{T}\left(\varphi \right)Qe\left(\varphi \right)d\varphi ,\\ V_{3}\left(i,t\right)= & \bar{d}\int_{-\bar{d}}^{0}\int_{t+\varphi }^{t}{\dot{e}}^{T}\left(\eta \right)R\dot{e}\left(\eta \right)d\eta d\varphi . \end{array}$$

The weak infinitesimal operator L of V(i,t) is defined in Equation (20):

(20) $$LV\left(i,t\right):=\lim_{\Delta \to 0}\frac{1}{\Delta }\left\{E\left\{V\left(\sigma \left(t+\Delta \right),t+\Delta \right)|\sigma \left(t\right)=i\right\}-V\left(i,t\right)\right\},$$

with

$$\begin{array}{cc} \; & \lim_{\Delta \to 0}\frac{1}{\Delta }\frac{G_{i}\left(h+\Delta \right)-G_{i}\left(h\right)}{1-G_{i}\left(h\right)}=0,\\ \; & \lim_{\Delta \to 0}\frac{1}{\Delta }\frac{1-G_{i}\left(h+\Delta \right)}{1-G_{i}\left(h\right)}=1,\\ \; & \lim_{\Delta \to 0}\frac{1}{\Delta }\frac{q_{ij}\left(G_{i}\left(h\right)-G_{i}\left(h+\Delta \right)\right)}{\Delta (1-G_{i}\left(h\right))}=\pi_{ij}\left(h\right), \end{array}$$

where *h* is the elapsed time at mode *i*, $G_{i}\left(h\right)$ represents the cumulative distribution function of the sojourn time, $q_{ij}$ denotes the probability intensity jumping from mode *i* to mode *j* and $\pi_{ij}\left(h\right):=q_{ij}\pi_{i}\left(h\right)$.

As a result, it can be deduced that

$$\begin{array}{ccc} LV_{1}\left(i,t\right) & = & \lim_{▵\to 0}\frac{1}{▵}[\sum_{j=1,j\neq i}^{N}\mathrm{Pr}\left\{\sigma \left(t+▵\right)=j|\sigma \left(t\right)=i\right\}e^{T}\left(t+▵\right)P_{j}e\left(t+▵\right)\\ & & +\mathrm{Pr}\left\{\sigma \left(t+▵\right)=i\left|\sigma (t)=i\right.\right\}e^{T}\left(t+▵\right)P_{i}e\left(t+▵\right)-e^{T}\left(t\right)P_{i}e\left(t\right)]\\ & & \lim_{▵\to 0}\frac{1}{▵}[\sum_{j=1,j\neq i}^{N}\frac{q_{ij}\left(G_{i}\left(h+▵\right)-G_{i}\left(h\right)\right)}{1-G_{i}\left(h\right)}e^{T}\left(t+▵\right)P_{j}e\left(t+▵\right)\\ & & +\frac{\left(G_{i}\left(h+▵\right)-G_{i}\left(h\right)\right)}{1-G_{i}\left(h\right)}e^{T}\left(t+▵\right)P_{i}e\left(t+▵\right)]-e^{T}\left(t\right)P_{i}e\left(t\right)\\ & = & {\dot{e}}^{T}\left(t\right)P_{i}e\left(t\right)+e^{T}\left(t\right)P_{i}\dot{e}\left(t\right)+\sum_{j=1}^{N}\pi_{ij}\left(h\right)e^{T}\left(t\right)P_{j}e\left(t\right)\\ & = & 2e^{T}\left(t\right)P_{i}\dot{e}\left(t\right)+\sum_{j=1}^{N}\pi_{ij}\left(h\right)e^{T}\left(t\right)P_{j}e\left(t\right)\\ & = & 2e^{T}\left(t\right)P_{i}({\bar{A}}_{i}e\left(t\right)+G_{i}\left(e\left(t\right)\right)-\left(F_{i}-K_{i}L_{i}\right)C_{i}e\left(t-d\left(t\right)\right)\\ & & +\left(F_{i}-K_{i}L_{i}\right)\epsilon \left(t-d\left(t\right)\right)+{\bar{B}}_{i}w_{1}\left(t\right)-\left(F_{i}-K_{i}L_{i}\right){\bar{D}}_{i}w_{2}\left(t-d(t)\right))\\ & & +\sum_{j=1}^{N}\pi_{ij}\left(h\right)e^{T}\left(t\right)P_{j}e\left(t\right) \end{array}$$

$$LV_{2}\left(i,t\right)=e^{T}\left(t\right)Qe\left(t\right)-e^{T}\left(t-\bar{d}\right)Qe\left(t-\bar{d}\right),$$

$$\begin{array}{ccc} LV_{3}\left(i,t\right) & = & \bar{d}\int_{-\bar{d}}^{0}{\dot{e}}^{T}\left(t\right)R\dot{e}\left(t\right)-\bar{d}\int_{-\bar{d}}^{0}{\dot{e}}^{T}\left(t+\varphi \right)R\dot{e}\left(t+\varphi \right)d\varphi \\ & = & {\bar{d}}^{2}{\dot{e}}^{T}\left(t\right)R\dot{e}\left(t\right)-\bar{d}\int_{t-\bar{d}}^{t}{\dot{e}}^{T}\left(\varphi \right)R\dot{e}\left(\varphi \right)d\varphi . \end{array}$$

In light of Lemma 1 as stated in Equation (18), one can obtain that

$$-\bar{d}\int_{t-\bar{d}}^{t}{\dot{e}}^{T}\left(\varphi \right)R\dot{e}\left(\varphi \right)d\varphi \leq {\left[\begin{array}{c} e^{T}\left(t\right)\\ e^{T}\left(t-d\left(t\right)\right)\\ e^{T}\left(t-\bar{d}\right) \end{array}\right]}^{T}\left[\begin{array}{ccc} -R & R & 0\\ {}* & -2R & R\\ {}* & * & -R \end{array}\right]\left[\begin{array}{c} e^{T}\left(t\right)\\ e^{T}\left(t-d\left(t\right)\right)\\ e^{T}\left(t-\bar{d}\right) \end{array}\right]$$

Moreover, from the Equation (16), one has

$${\bar{d}}^{2}{\dot{e}}^{T}\left(t\right)R\dot{e}\left(t\right)=\eta^{T}\left(t\right)\left[\begin{array}{c} \bar{d}{\bar{A}}_{i}^{T}\\ -\bar{d}C_{i}^{T}{\left(F_{i}-K_{i}L_{i}\right)}^{T}\\ 0\\ \bar{d}{\left(F_{i}-K_{i}L_{i}\right)}^{T}\\ \bar{d}I\\ \bar{d}{\bar{B}}_{i}^{T}\\ -\bar{d}{\bar{D}}_{i}^{T}{\left(F_{i}-K_{i}L_{i}\right)}^{T} \end{array}\right]R{\left[\begin{array}{c} \bar{d}{\bar{A}}_{i}^{T}\\ -\bar{d}C_{i}^{T}{\left(F_{i}-K_{i}L_{i}\right)}^{T}\\ 0\\ \bar{d}{\left(F_{i}-K_{i}L_{i}\right)}^{T}\\ \bar{d}I\\ \bar{d}{\bar{B}}_{i}^{T}\\ -\bar{d}{\bar{D}}_{i}^{T}{\left(F_{i}-K_{i}L_{i}\right)}^{T} \end{array}\right]}^{T}\eta \left(t\right)$$

where

$$\eta \left(t\right)={\left[\begin{array}{ccccccc} e^{T}\left(t\right) & e^{T}\left(t-d_{k}\left(t\right)\right) & e^{T}\left(t-\bar{d}\right) & \epsilon^{T}\left(t-d\left(t\right)\right) & G_{i}^{T}\left(e\left(t\right)\right) & w_{1}^{T}\left(t\right) & w_{2}^{T}\left(t-d(t)\right) \end{array}\right]}^{T}$$

From the definition of nonlinear function $G_{i}$ as stated in Equation (1), it yields that

$$G_{i}^{T}\left(e\left(t\right)\right)G_{i}\left(e\left(t\right)\right)\leq \Gamma_{i}e^{T}\left(t\right)e\left(t\right),$$

which means that

$$\Gamma_{i}e^{T}\left(t\right)e\left(t\right)-G_{i}^{T}\left(e\left(t\right)\right)G_{i}\left(e\left(t\right)\right)\geq 0.$$

The event-triggering function in Equation (10) implies that

$$\sum_{m=1}^{N}\epsilon_{m}^{T}\left(t_{k}\right)\epsilon_{m}\left(t_{k}\right)\leq \sum_{m=1}^{N}\kappa_{i}^{2}{\left({\tilde{y}}_{m}\left(t_{k}\right)-\epsilon_{i}\left(t_{k}\right)\right)}^{T}\left({\tilde{y}}_{m}\left(t_{k}\right)-\epsilon_{m}\left(t_{k}\right)\right)$$

which can lead to

$${\left[\begin{array}{c} e(t-d(t))\\ \epsilon (t-d(t)) \end{array}\right]}^{T}\left[\begin{array}{cc} C_{i}^{T}\left(\Lambda_{i}⊗I\right)C_{i} & -C_{i}^{T}\left(\Lambda_{i}⊗I\right)\\ {}* & (\Lambda_{i}-I)⊗I \end{array}\right]\left[\begin{array}{c} e(t-d(t))\\ \epsilon (t-d(t)) \end{array}\right]\geq 0,$$

where $\Lambda_{i}=diag\left\{\kappa_{1}^{2},\kappa_{2}^{2},\ldots ,\kappa_{N}^{2}\right\}.$

Then, it can be obtained by Schur complement that

$$\begin{array}{ccc} & & LV\left(i,t\right)+\Gamma_{i}e^{T}\left(t\right)e\left(t\right)-G_{i}^{T}\left(e\left(t\right)\right)G_{i}\left(e\left(t\right)\right)\\ & & -2e^{T}\left(t\right){\bar{E}}_{i}^{T}S\left(w_{1}\left(t\right)+w_{2}\left(t-d(t)\right)\right)-\gamma w_{1}^{T}\left(t\right)w_{1}\left(t\right)-\gamma w_{2}^{T}\left(t-d(t)\right)w_{2}\left(t-d(t)\right)\\ & & +{\left[\begin{array}{c} e(t-d(t))\\ \epsilon (t-d(t)) \end{array}\right]}^{T}\left[\begin{array}{cc} C_{i}^{T}\left(\Lambda_{i}⊗I\right)C_{i} & -C_{i}^{T}\left(\Lambda_{i}⊗I\right)\\ {}* & (\Lambda_{i}-I)⊗I \end{array}\right]\left[\begin{array}{c} e(t-d(t))\\ \epsilon (t-d(t)) \end{array}\right]\\ & \leq & \eta^{T}\left(t\right){\tilde{\Xi }}_{i}\eta \left(t\right), \end{array}$$

where

$$\begin{array}{cc} {\tilde{\Xi }}_{i}: & =\left[\begin{array}{cc} {\tilde{\Xi }}_{1i} & {\tilde{\Xi }}_{2i}\\ {}* & {\tilde{\Xi }}_{3i} \end{array}\right],\\ {\tilde{\Xi }}_{1i}: & =\left[\begin{array}{ccc} 2P_{i}{\bar{A}}_{i}+Q-R+\Gamma_{i}+\sum_{j=1}^{N}\pi_{ij}\left(h\right)P_{j} & -P_{i}\left(F_{i}-K_{i}L_{i}\right)C_{i}+R & 0\\ {}* & -2R+C^{T}\left(\Lambda ⊗I_{q}\right)C & R\\ {}* & * & -Q-R \end{array}\right],\\ {\tilde{\Xi }}_{2i}: & =\left[\begin{array}{ccccc} P_{i}\left(F_{i}-K_{i}L_{i}\right) & P_{i} & P_{i}{\bar{B}}_{i}-2{\bar{E}}_{i}^{T} & -P_{i}\left(F_{i}-K_{i}L_{i}\right){\bar{D}}_{i}-2{\bar{E}}_{i}^{T}S & \bar{d}{\bar{A}}_{i}^{T}\\ -C^{T}\left(\Lambda ⊗I_{q}\right) & 0 & 0 & 0 & -\bar{d}C_{i}^{T}{\left(F_{i}-K_{i}L_{i}\right)}^{T}\\ 0 & 0 & 0 & 0 & 0 \end{array}\right],\\ {\tilde{\Xi }}_{3i}: & =\left[\begin{array}{ccccc} \left(\Lambda -I_{N}\right)⊗I_{q} & 0 & 0 & 0 & \bar{d}{\left(F_{i}-K_{i}L_{i}\right)}^{T}\\ {}* & -I & 0 & 0 & \bar{d}I\\ {}* & * & -\gamma I & 0 & \bar{d}{\bar{B}}_{i}^{T}\\ {}* & * & * & -\gamma I & -\bar{d}{\bar{D}}_{i}^{T}{\left(F_{i}-K_{i}L_{i}\right)}^{T}\\ {}* & * & * & * & -R^{-1} \end{array}\right]. \end{array}$$

By performing congruent transformation to ${\tilde{\Xi }}_{i}$ by diag{I,I,I,I,I,I,I,R},$\Xi_{i}$ can be obtained. Therefore, it can be verified that when $\Xi_{i}<0$ holds, the following inequality holds by integrating between 0 and *T* that

(21) $$2E\left\{\int_{0}^{T}{\tilde{z}}^{T}\left(t\right)S\left(w_{1}\left(t\right)+w_{2}\left(t-d(t)\right)\right)dt\right\}+\gamma \int_{0}^{T}w_{1}^{T}\left(t\right)w_{1}\left(t\right)+w_{2}^{T}\left(t-d(t)\right)w_{2}\left(t-d(t)\right)dt\geq 0.$$

Finally, by taking into account the time-varying dwell time h(t) [28], one has $\pi_{ij}\left(h\right)=\sum_{1}^{K}\lambda_{k}\pi_{ij,k}$, $\sum_{1}^{K}\lambda_{k}=1$, $\lambda_{k}\geq 0$. This implies that if $\Xi_{i,k}<0$ holds, the passivity performance can be achieved according to Definition 1 in the mean-square sense, which completes the proof. □

**Theorem** **2.** *For given $h_{k}$, the passivity filtering can be achieved according to Definition 1, if there exist mode-dependent matrices $P_{i}>0$, ${\tilde{F}}_{i}$ and ${\tilde{K}}_{i}$, mode-dependent parameters $\kappa_{i}$, and matrices Q>0, R>0, such that $\Theta_{i,k}<0$, for all i∈N, k=1,2,…,K, where*

$$\begin{array}{cc} \Theta_{i,k}: & =\left[\begin{array}{cc} \Theta_{1i,k} & \Theta_{2i,k}\\ {}* & \Theta_{3i,k} \end{array}\right],\\ \Theta_{1i,k}: & =\left[\begin{array}{cccc} 2P_{i}{\bar{A}}_{i}+Q-R+\Gamma_{i}+\sum_{j=1}^{N}\pi_{ij,k}P_{j} & -{\tilde{F}}_{i}C_{i}+{\tilde{K}}_{i}L_{i}C_{i}+R & 0 & {\tilde{F}}_{i}-{\tilde{K}}_{i}L_{i}\\ {}* & -2R+C^{T}\left(\Lambda ⊗I_{q}\right)C & R & -C^{T}\left(\Lambda ⊗I_{q}\right)\\ {}* & * & -Q-R & 0\\ {}* & * & * & \left(\Lambda -I_{N}\right)⊗I_{q} \end{array}\right],\\ \Theta_{2i,k}: & =\left[\begin{array}{cccc} P_{i} & P_{i}{\bar{B}}_{i}-{\bar{E}}_{i}^{T}S & -{\tilde{F}}_{i}{\bar{D}}_{i}+{\tilde{K}}_{i}L_{i}{\bar{D}}_{i}-{\bar{E}}_{i}^{T} & \bar{d}{\bar{A}}_{i}^{T}P_{i}\\ 0 & 0 & 0 & -\bar{d}C_{i}^{T}{\tilde{F}}_{i}^{T}+\bar{d}C_{i}^{T}L_{i}^{T}{\tilde{K}}_{i}^{T}\\ 0 & 0 & 0 & 0\\ 0 & 0 & 0 & \bar{d}{\tilde{F}}_{i}^{T}-\bar{d}L_{i}^{T}{\tilde{K}}_{i}^{T} \end{array}\right],\\ \Theta_{3i,k}: & =\left[\begin{array}{cccc} -I & 0 & 0 & \bar{d}P_{i}\\ {}* & -\gamma I & 0 & \bar{d}{\bar{B}}_{i}^{T}P_{i}\\ {}* & * & -\gamma I & -\bar{d}{\bar{D}}_{i}^{T}{\tilde{F}}_{i}^{T}+\bar{d}{\bar{D}}_{i}^{T}L_{i}^{T}{\tilde{K}}_{i}^{T}\\ {}* & * & * & R-2P_{i} \end{array}\right]. \end{array}$$

*With the above feasible solutions, the mode-dependent filter gains $K_{i}$ and $F_{i}$ can be calculated by*

$$\begin{array}{cc} F_{i}= & P_{i}^{-1}{\tilde{F}}_{i},\\ K_{i}= & P_{i}^{-1}{\tilde{K}}_{i}. \end{array}$$

**Proof.**  Based on matrix transformation, the proof can follow directly from Theorem 1. □

**Remark** **3.** *It is worth mentioning that the derived conditions are in the form of strict LMIs, which can be easily solved by Matlab or other mathematical convex optimization softwares. Once the feasible solution is solved, then the corresponding filter gains can be obtained.*

## 4. Illustrative Example

In this section, the simulation results are provided to validate the effectiveness of our derived results.

For the illustrative example, a numerical simulation is carried out in Matlab 2017a with numerical parameters.

Consider the flexible manipulator with following semi-Markov parameters according to system (3):

$$\begin{array}{cc} A_{1}= & \left[\begin{array}{cccc} 0 & 1 & 0 & 0\\ -50 & -1.25 & 50 & 0\\ 0 & 0 & 0 & 1\\ 10 & 0 & -10 & 0 \end{array}\right],\\ g_{1}\left(x\left(t\right)\right)= & \left[\begin{array}{c} 0\\ 0\\ 0\\ -5\sin(\theta_{1}) \end{array}\right],\\ B_{1}= & \left[\begin{array}{c} 0\\ 5\\ 0\\ 0 \end{array}\right],\\ E_{1} & =\left[\begin{array}{cccc} 1 & 0 & 0 & 0\\ 0 & 1 & 0 & 0 \end{array}\right]. \end{array}$$

and

$$\begin{array}{cc} A_{2}= & \left[\begin{array}{cccc} 0 & 1 & 0 & 0\\ -25 & -0.625 & 25 & 0\\ 0 & 0 & 0 & 1\\ 5 & 0 & -5 & 0 \end{array}\right],\\ g_{2}\left(x\left(t\right)\right)= & \left[\begin{array}{c} 0\\ 0\\ 0\\ -4\sin(\theta_{1}) \end{array}\right],\\ B_{2}= & \left[\begin{array}{c} 0\\ 2.5\\ 0\\ 0 \end{array}\right],\\ E_{2} & =\left[\begin{array}{cccc} 1 & 0 & 0 & 0\\ 0 & 1 & 0 & 0 \end{array}\right]. \end{array}$$

**Figure 2:**
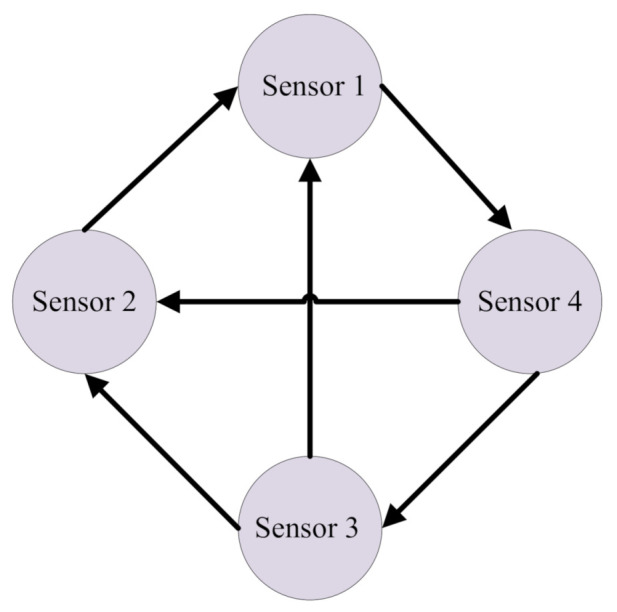
The topology of sensor network.

The sensor network consisted of 4 sensors are use with following parameters according to system (8) and its communication topology is depicted in Figure 2,

$$\begin{array}{cc} C_{11}= & \left[\begin{array}{cccc} 1.2 & 0 & 0 & 0\\ 0 & 1.2 & 0 & 0 \end{array}\right],D_{11}=\left[\begin{array}{c} 0.5\\ 0.5 \end{array}\right],\\ C_{21}= & \left[\begin{array}{cccc} 1.1 & 0 & 0 & 0\\ 0 & 1.1 & 0 & 0 \end{array}\right],D_{21}=\left[\begin{array}{c} 0.6\\ 0.6 \end{array}\right],\\ C_{31}= & \left[\begin{array}{cccc} 1.3 & 0 & 0 & 0\\ 0 & 1.3 & 0 & 0 \end{array}\right],D_{31}=\left[\begin{array}{c} 0.4\\ 0.4 \end{array}\right],\\ C_{41}= & \left[\begin{array}{cccc} 1.4 & 0 & 0 & 0\\ 0 & 1.4 & 0 & 0 \end{array}\right],D_{41}=\left[\begin{array}{c} 0.3\\ 0.3 \end{array}\right], \end{array}$$

and

$$\begin{array}{cc} C_{12}= & \left[\begin{array}{cccc} 0.9 & 0 & 0 & 0\\ 0 & 0.9 & 0 & 0 \end{array}\right],D_{12}=\left[\begin{array}{c} 0.2\\ 0.2 \end{array}\right],\\ C_{22}= & \left[\begin{array}{cccc} 0.8 & 0 & 0 & 0\\ 0 & 0.8 & 0 & 0 \end{array}\right],D_{22}=\left[\begin{array}{c} 0.4\\ 0.3 \end{array}\right],\\ C_{32}= & \left[\begin{array}{cccc} 0.7 & 0 & 0 & 0\\ 0 & 0.7 & 0 & 0 \end{array}\right],D_{32}=\left[\begin{array}{c} 0.6\\ 0.4 \end{array}\right],\\ C_{42}= & \left[\begin{array}{cccc} 0.6 & 0 & 0 & 0\\ 0 & 0.6 & 0 & 0 \end{array}\right],D_{42}=\left[\begin{array}{c} 0.7\\ 0.6 \end{array}\right], \end{array}$$

where the Laplacian can obtained as follows:

$$L=\left[\begin{array}{cccc} 2 & -1 & -1 & 0\\ 0 & 2 & -1 & -1\\ 0 & 0 & 1 & -1\\ -1 & 0 & 0 & 1 \end{array}\right]$$

Based on the above directed communication topology, sensor nodes 1–4 can exchange information accordingly. It is noticed that all sensors are with different parameters to verify the general applicability of our filtering design. In practical applications, the adopted sensor networks can also be chosen with same parameters.

In the simulation, the sampling period is randomly set by 0.1 s and 0.2 s, such that $\bar{d}=0.2$ s. The transition rates are supposed to be $\pi_{11}\left(h\right)\in \left[-1.6,-1.4\right]$ and $\pi_{22}\left(h\right)\in \left[-1.9,-1.1\right]$, which implies that $\pi_{11,1}=-1.4$, $\pi_{11,2}=-1.6$, $\pi_{22,1}=-1.1$ and $\pi_{22,2}=-1.9$ with K=2. Moreover, the mode-dependent event-triggered scalars are supposed to be $\kappa_{1}=0.1$ and $\kappa_{2}=0.2$. The passivity performance index is chosen by γ=10 and the disturbances are set by $w_{1}\left(t\right)=0.1sin\left(t\right)$ and $w_{2}\left(t\right)=0.1cos\left(t\right)$. With these parameters, the desired mode-dependent filter gains can be obtained by

$$\begin{array}{ccc} K_{11} & = & \left[\begin{array}{cc} -0.0631 & -0.0022\\ -0.2574 & -0.0358\\ -0.0315 & -0.0021\\ 0.0361 & 0.0063 \end{array}\right],\\ K_{21} & = & \left[\begin{array}{cc} -0.0611 & -0.0020\\ -0.2371 & -0.0285\\ -0.0299 & -0.0014\\ 0.0321 & 0.0050 \end{array}\right],\\ K_{31} & = & \left[\begin{array}{cc} -0.0884 & -0.0024\\ -0.2714 & -0.0296\\ -0.0409 & -0.0016\\ 0.0338 & 0.0056 \end{array}\right],\\ K_{41} & = & \left[\begin{array}{cc} -0.0988 & -0.0024\\ -0.2703 & -0.0397\\ -0.0437 & -0.0027\\ 0.0336 & 0.0076 \end{array}\right], \end{array}$$

and

$$\begin{array}{cc} K_{12}= & \left[\begin{array}{cc} 0.0236 & -0.0022\\ -0.1970 & 0.0037\\ -0.0069 & 0.0007\\ 0.0318 & -0.0019 \end{array}\right],\\ K_{22}= & \left[\begin{array}{cc} 0.0114 & -0.0060\\ -0.2303 & 0.0010\\ -0.0149 & -0.0006\\ 0.0354 & -0.0017 \end{array}\right],\\ K_{32}= & \left[\begin{array}{cc} 0.0248 & -0.0108\\ -0.2609 & -0.0118\\ -0.0156 & -0.0029\\ 0.0395 & -0.0005 \end{array}\right],\\ K_{42}= & \left[\begin{array}{cc} 0.0471 & -0.0007\\ -0.1247 & 0.0021\\ 0.0021 & 0.0010\\ 0.0203 & -0.0020 \end{array}\right], \end{array}$$

and

$$\begin{array}{cc} F_{11}= & \left[\begin{array}{cc} 0.0940 & -0.0068\\ -0.9188 & 0.3561\\ -0.0043 & 0.0158\\ 0.1647 & -0.0651 \end{array}\right],\\ F_{21}= & \left[\begin{array}{cc} 0.0761 & -0.0081\\ -0.9721 & 0.3893\\ -0.0110 & 0.0175\\ 0.1716 & -0.0710 \end{array}\right],\\ F_{31}= & \left[\begin{array}{cc} 0.1059 & -0.0044\\ -0.7079 & 0.3739\\ 0.0093 & 0.0172\\ 0.1277 & -0.0674 \end{array}\right],\\ F_{41}= & \left[\begin{array}{cc} 0.1148 & -0.0029\\ -0.5616 & 0.3428\\ 0.0202 & 0.0150\\ 0.1046 & -0.0615 \end{array}\right], \end{array}$$

and

$$\begin{array}{cc} F_{12}= & \left[\begin{array}{cc} 1.0483 & 0.0050\\ -0.7521 & 0.5298\\ 0.3404 & 0.0511\\ 0.2321 & -0.0889 \end{array}\right],\\ F_{22}= & \left[\begin{array}{cc} 1.0430 & -0.0045\\ -1.0643 & 0.5668\\ 0.3113 & 0.0520\\ 0.2824 & -0.0958 \end{array}\right],\\ F_{32}= & \left[\begin{array}{cc} 1.0082 & -0.0081\\ -1.1269 & 0.6160\\ 0.2850 & 0.0549\\ 0.2896 & -0.1038 \end{array}\right],\\ F_{42}= & \left[\begin{array}{cc} 1.0100 & -0.0047\\ -1.3132 & 0.6969\\ 0.2634 & 0.0643\\ 0.3202 & -0.1174 \end{array}\right]. \end{array}$$

**Figure 3:**
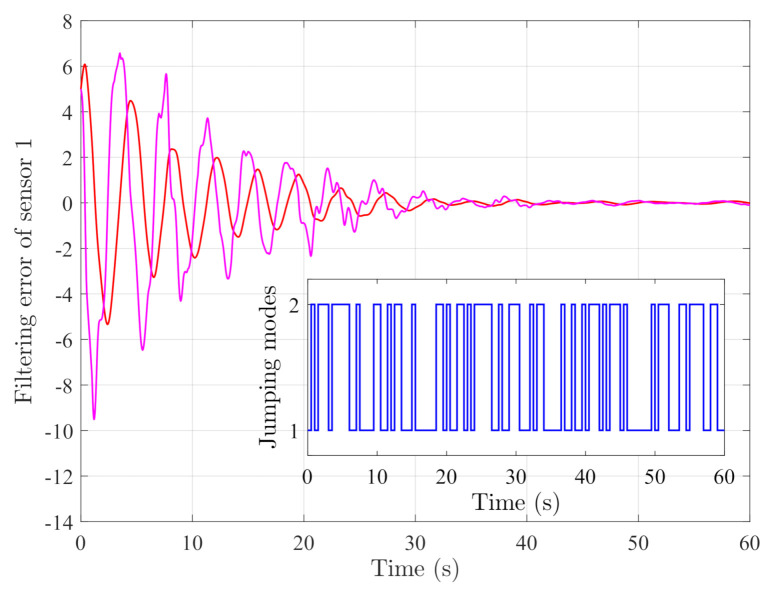
Filtering errors of sensor 1.

**Figure 4:**
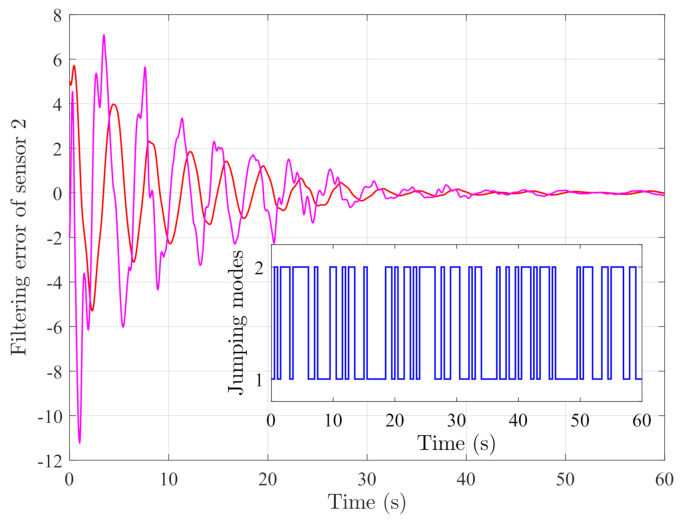
Filtering errors of sensor 2.

**Figure 5:**
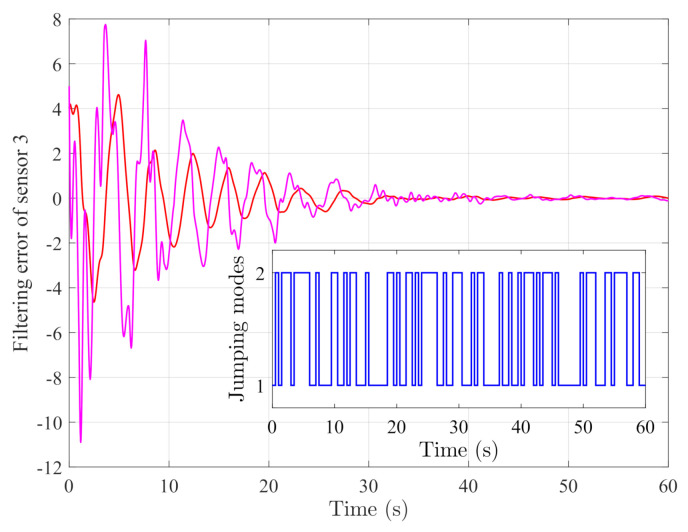
Filtering errors of sensor 3.

**Figure 6:**
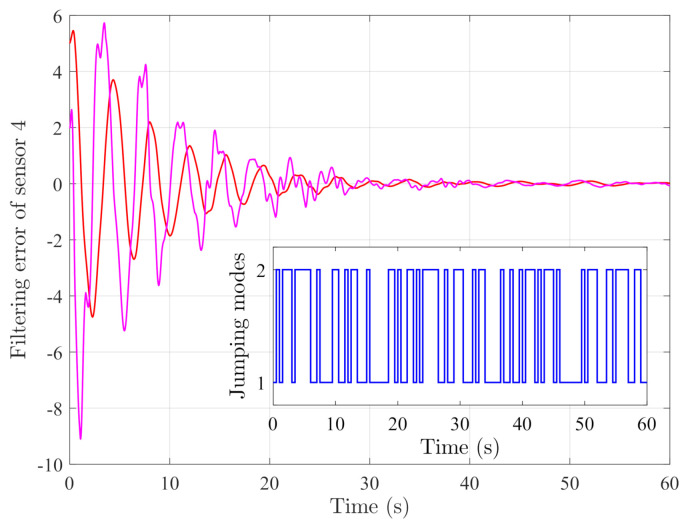
Filtering errors of sensor 4.

**Figure 7:**
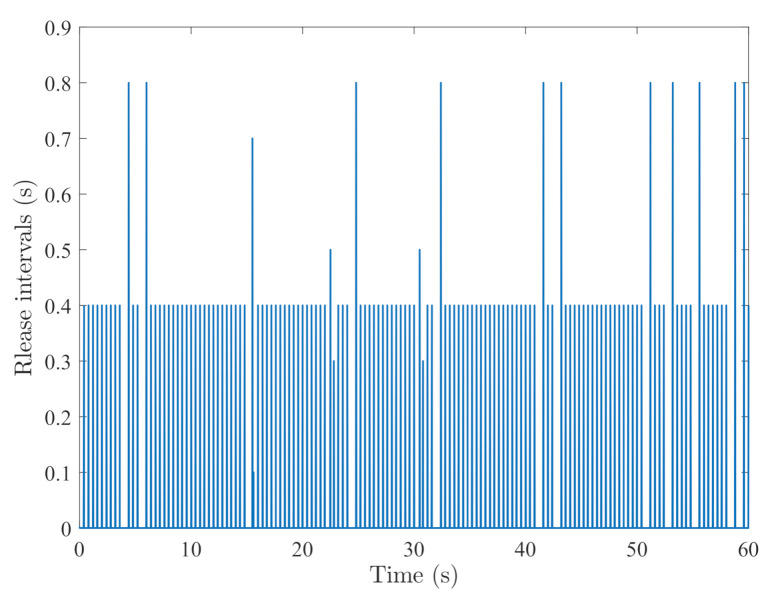
Event-triggered instants of sensor 1.

**Figure 8:**
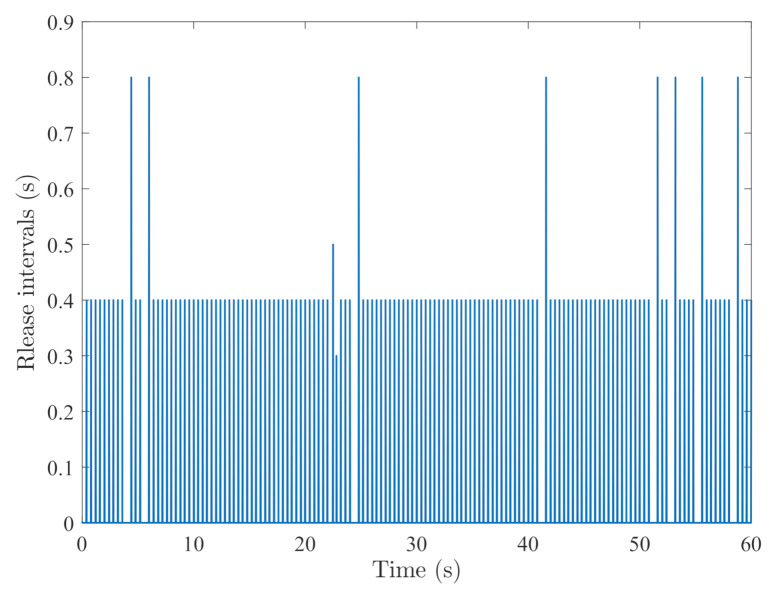
Event-triggered instants of sensor 2.

**Figure 9:**
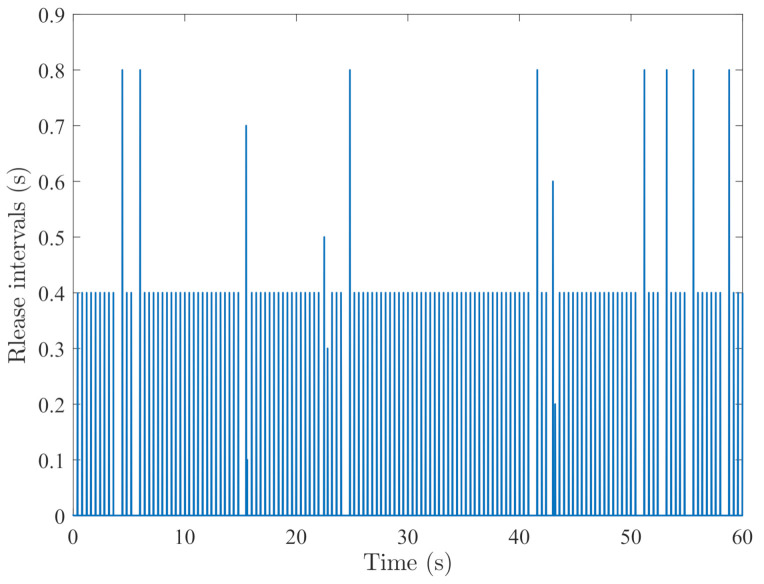
Event-triggered instants of sensor 3.

**Figure 10:**
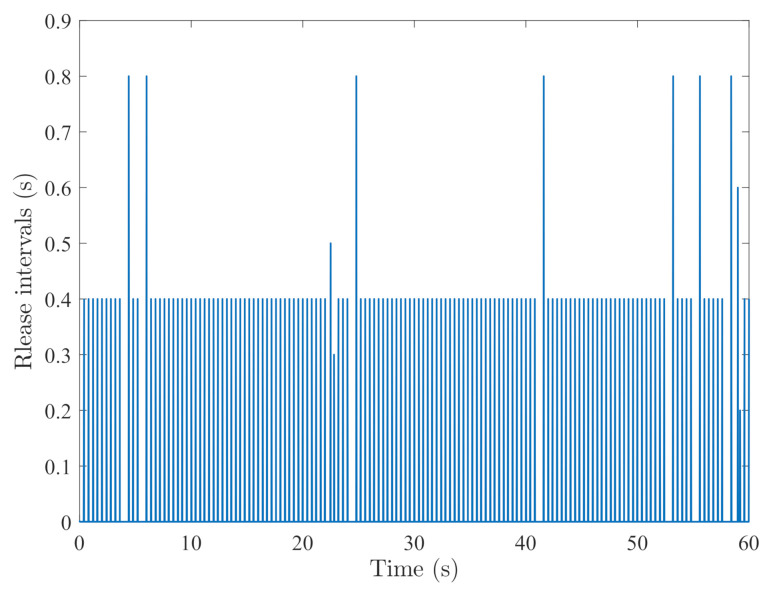
Event-triggered instants of sensor 4.

By random initial conditions for sensor nodes, Figure 3, Figure 4, Figure 5 and Figure 6 show the state trajectories of the filtering errors with disturbances. It can be seen that all the filtering errors can converge to zeros despite of system mode jumping and external disturbances, which means that the designed mode-dependent filters can effectively estimate the true state of flexible manipulator according to Definition 1. This also implies that the filtering errors $e_{m}\left(t\right)=x\left(t\right)-{\hat{x}}_{m}\left(t\right)$ for all sensors can be mean-square stable when there is no disturbance, which can verify our derived results. Figure 7, Figure 8, Figure 9 and Figure 10 depict event triggering instants and release interval of the sensors, where the release intervals are larger and the signal transmission among the sensors is event-triggered instead of traditional time-triggered schemes. It can be found that the developed event-triggered strategy can considerably decrease the numbers of communications compared with tradition time-triggered schemes (time-varying sampling periods of 0.1 s and 0.2 s). This shows the considerable advantages on decreasing signal transmissions among the sensor networks, where the event-triggered instants are larger than the tradition time-triggered instants. Thus, the simulation results can support our developed filter designs.

## 5. Conclusions

This paper discusses distributed filters design of flexible manipulator with semi-Markov parameters based on sensor networks. Moreover, the passivity performance is adopted to cope with the external disturbances of manipulator and sensor networks. By developing mode-dependent event-triggered schemes to achieve information exchanges, the distributed filtering can be accomplished in an asynchronous framework. By employing mode-dependent Lyapunov–Krasovskii approach, sufficient filtering conditions can be deduced and desired filter gains are designed, such that the passivity performance can be achieved. The correctness of our design method is finally demonstrated by a numerical example. In the future study, an interesting issue would be extending our current results to the cases with sampled semi-Markov processes, which means that the observed jumping modes may be different from the true system modes, which is more complex but more practical in the real world applications.

## Data Availability

Not applicable.
